# Recycling of waste coffee grounds as a photothermal material modified with ZnCl_2_ for water purification

**DOI:** 10.1038/s41598-024-61768-y

**Published:** 2024-05-11

**Authors:** Elias Wagari Gabisa, Chavalit Ratanatamskul

**Affiliations:** 1 Department of Environmental Engineering, Chulalongkorn University, Bangkok, Thailand; 2 Center of Excellence in Innovative Waste Treatment and Water Reuse, Faculty of Engineering, Chulalongkorn University, Bangkok, 10330 Thailand; 3 Bahir Dar Institute of Technology, Faculty of Chemical and Food Engineering, Bahir Dar University, Bahir Dar, Ethiopia

**Keywords:** Waste coffee ground, Water, Purification, Carbonization, Photothermal material, ZnCl_2_, Environmental sciences, Materials for energy and catalysis

## Abstract

The aim of this study was to develop a photothermal material modified with carbonization and ZnCl_2_ impregnation and supported by polyvinyl alcohol (PVA) for water purification using the waste coffee grounds. Scanning electron microscopy (SEM) characterization of the prepared material revealed that a significant surface modification was achieved due to the carbonization and ZnCl_2_ impregnation. X-ray diffraction analysis (XRD) pattern of the samples showed two broad peaks at 18.4° and 22.2°, this is due to the crystal planes of β-crystal phase structure, which indicates the existence of strong hydrogen bonds between the micro-structures and therefore less suspectable to chemical attack. Additionally, thermogravimetric analysis (TGA) result suggests a slight mass reduction between the temperature range of 65–75 °C implying the thermal stability of the prepared material. The produced modified material had a photothermal conversion efficiency of 74% and could produce vapor at a rate of 1.12 kg/m^2^h under 980 W/m^2^ irradiation at 1 sun. A significant reduction in Cu^2+^ ion concentration (83%), turbidity (91%), total dissolved solids (TDS) (61%), microbial load (95.6%), and total hardness (41.2%) were achieved. Therefore, waste coffee grounds can be considered as a future eco-friendly and low-cost candidate for water purification.

## Introduction

Water scarcity is one of the current issues that affect human and other living things worldwide. Researchers and other organizations around the world have therefore devoted a lot of attention to this issue^1,2^. Even though the majority of the earth’s surface is covered by water (67%), the amount available for human utilization is very low^3^. Numerous studies have been conducted to address the problems with desalination and water purification technology, which includes membrane desalination^4^, semi-permeable reverse osmosis^5^ and electro-dialysis reversal^6^ in order to provide drinkable water. However, the economic viability of such technologies in remote areas and developing countries has been questioned due to their higher energy consumption^7^. There are attempts to purify lake water for rural and urban consumption as well. For instance, the study conducted using sand, rice husk, coconut fiber, and activated carbon revealed that it is possible to remove pollutants from lake water^8^. An integrated water treatment method has also been employed to treat lake water, and they found that improvements were observed in terms of metal ion removal as well as organic constituents^9^.

Solar energy is a freely available energy source for everyone and has recently been researched for water purification. It is preferred because it is environmentally friendly with minimal impact. However, the solar-water treatment process has shortcomings like vapor conversion at low light, which limits the applicability of the system. Process losses and lower light absorption for water purification are also among the limitations^10–12^. To address these shortcomings, various photothermal materials (PTMs) have been formulated to enhance the solar light absorption performance and solar thermal conversion efficiency^13–18^. Photothermal materials have emerged as a promising class of materials for applications like solar energy conversion and water desalination. Their ability to absorb light and convert it into heat makes them valuable tools for a variety of technologies. Research efforts in PTM development focus on both synthesis methods and material design. On the synthesis side, there is a drive to create PTMs using techniques that are cost-effective, scalable, and environmentally friendly. Mechanochemical synthesis is a recent development that shows promise in this regard^19^. Material design strategies focus on maximizing light absorption efficiency and controlling thermal properties to optimize heat transfer for the desired application. For example, in solar steam generation, researchers target PTMs with strong solar light absorption and minimal heat loss to the environment^20^. By considering both synthesis and design, researchers are creating increasingly effective PTMs.

Solar steam generation has emerged as a promising technology for sustainable applications like desalination, sterilization, and industrial process heating. It harnesses sunlight to heat water and produce steam, offering a clean alternative to fossil fuels and reducing greenhouse gas emissions^19^. Traditionally, this technology relied on concentrated solar power (CSP) systems. Parabolic troughs or dishes focus sunlight onto a receiving vessel containing water, achieving high temperatures but incurring significant costs due to the complex concentrating infrastructure^21^. Recent advancements explore alternative designs, including flat-panel collectors with photothermal materials (PTMs) incorporated onto the absorber surface. PTMs efficiently absorb sunlight and convert it to heat, promoting water evaporation and steam generation. This approach offers a simpler and potentially more cost-effective solution for various applications^21^. Ongoing research focuses on optimizing PTM design to maximize light absorption, minimize heat loss, and achieve efficient steam generation under varying operating conditions^19,22^. Additionally, researchers are exploring integrating these systems with nanotechnology for further efficiency improvements^22^.

For instance, a study conducted to enhance efficient solar thermal generation revealed that Mxenes material could improve the structure of the material, thereby improving its hydrophobicity^23^. Other studies also employed hydrogel to enhance the sorption capacity of deliquescent salt^24^. Perovskite oxide modified photothermal membrane showed enhanced energy efficiency as well as better steam generation^25^. In-situ polymerized MnO_2_@ppy nanocomposites also performed well and showed an increase in thermal steam generation^26^.

Nanostructured metals have also been investigated to increase the solar absorption intensity and solar thermal conversion efficiency of materials. For example, high efficiency solar thermal conversion was achieved by preparing a bilayer Janus film through the incorporation of gold nanorods in carbon nanotubes^27^. Higher evaporation efficiency was obtained with plasmonic active filter paper modified by gold nanoparticles^28^.

Recently, there have been emerging studies considering carbon-based materials for better solar vaporization^29–33^. For instance, salt resistant carbon dots have been developed and modified for desalination and steam power generation^21^. Coffee is among the most consumed beverages in the world and the second-most valuable commodity after petroleum. The coffee industry generates a high amount of waste residue, which has almost no use in many of the developing countries^34^. During the coffee brewing process, the only intention is to extract the very important components from the bean, which is very small. The remaining coffee grounds are leftover, which is almost equal to the amount of coffee consumed. According to the International Coffee Organization, coffee consumption reached 166 million 60-kg bags in 2021. Globally, about 6 million tons of spent coffee grounds are produced annually^30^. For every gram of coffee produced, 0.91 g of waste coffee grounds are produced^35^. Waste coffee grounds have high oxygen consumption during the decomposition process and the presence of phenols, caffeine, and tannins all of which are extremely toxic to numerous life processes makes it an extremely dangerous pollutant^36,37^. However, these waste coffee grounds are cellulosic materials, which makes it very convenient to produce carbon materials for different applications^38^. Therefore, waste coffee grounds have been getting attention recently and are being applied for different purposes.

Different studies have considered different biomass residues (corn cob, coffee husk, peanut hulls, cotton stocks etc.) as a precursor for activated carbon production^39–42^. Interestingly, waste coffee ground has low ash content and high carbon content, which makes its carbonization process seamless and efficient. However, waste coffee ground (WCG) is rarely considered for such purposes. These limited studies considered just carbonization to activate the surface of the waste coffee ground. In this study, we have proposed a novel three-step modification approach, which is carbonization followed by ZnCl_2_ activation and poly vinyl alcohol modification. Salts like ZnCl_2_ can affect the textural parameters as well as the surface chemistry of carbonized biomass^43^. For instance, a modification of bamboo biochar with K_3_PO_4_ for six hours has changed the surface significantly^44^. The other study considered ZnCl_2_ on biochar prepared from Yunnan pine powder and the surface has been significantly improved^45^. Furthermore, the reduced ZnCl_2_ can interact with other non-bonding electrons of oxygen atom to form Zn–O. This leads to better surface chemistry and, hence, pollutant adsorption^46^.

Therefore, the idea of preparing a functional photothermal material for the treatment of lake water using the abundant WCG is proposed in this research. The production of a bio-based photothermal material can help to use the discarded WCG. This idea properly aligned to the concepts of zero-waste practice and bio-circular economy in society. At present, there is little research work on the utilization/modification of the WCG as a photothermal material. Thus, this study proposed and evaluated a novel modification method for activating the WCG to achieve a photothermal material with high efficacy. Here, a novel three-step modification approach—carbonization followed by ZnCl_2_ activation and poly vinyl alcohol modification—is introduced. The modified WCG (MCWCG) was characterized and experimentally tested to ascertain its efficacy, and the test results show that the MCWCG can provide higher excellent efficacy. Furthermore, using the amount of WCG and PVA concentration as experimental factors and evaporation rate as a response was also investigated. Water purification process was also optimized using response surface methodology. Optimum conditions to achieve the highest water purification were established. Finally, the produced material of modified carbonized waste coffee ground (MCWCG) was characterized, and experimentally tested and validated by using it to treat the simulated lake water.

## Materials and methods

### Preparation of ZnCl_2_ and PVA modified carbonized coffee ground

**Figure 1:**
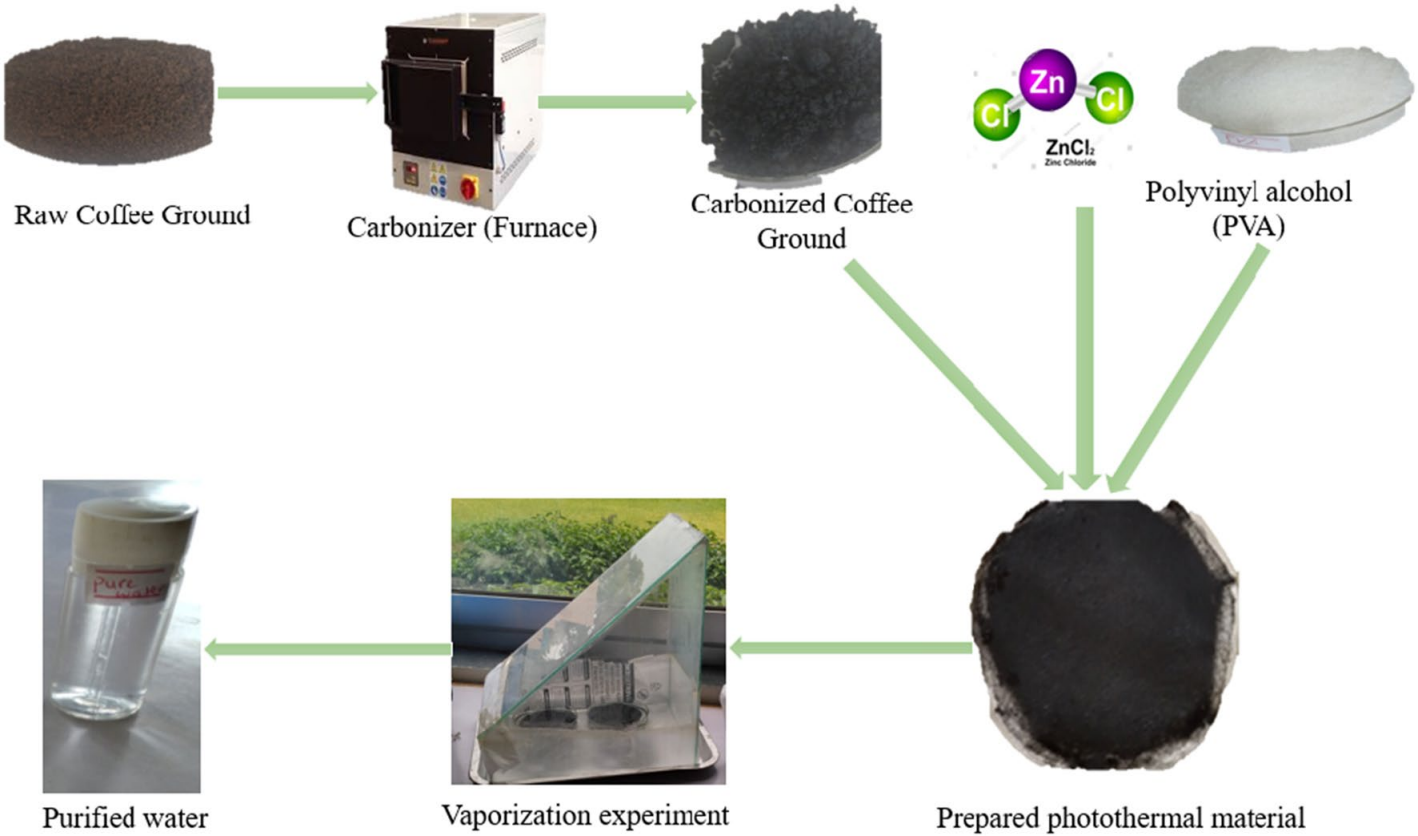
Schematic diagram of a novel photothermal material preparation.

The schematic diagram of the novel photomaterial material is shown in Fig. 1. The synthesis design of photothermal materials involves meticulous selection of light absorbers and matrices, followed by tailored fabrication techniques. Light absorbers, often nanoparticles or organic molecules, determine the material's wavelength selectivity and efficiency. Waste coffee grounds were collected from the neighborhood coffee establishments. The waste coffee grounds was washed and allowed to dry at 105° C for overnight. The dried coffee ground was then placed in a crucible and covered with aluminum foil. Then it was placed in a nitrogen induced furnace and carbonized at 900 °C for 1 h at a heating rate of 5 °C/min. The carbonized waste coffee ground was allowed to cool. The carbonized waste coffee ground (CWCG) was then impregnated with ZnCl_2_ at a ratio of 1 g to 10 mL of 1 M ZnCl_2_.

This proposed simultaneous process can enhance the availability of pores and channels in the resulting CWCG with sizes and shapes by the salt crystals. Additionally, ZnCl_2_ leaves metal and/or metal oxide residues after decomposition during carbonization, which introduces catalytic sites and other functionalities to the material. Polyvinyl alcohol (PVA) was selected as a support because of its availability and good hydrophobicity. The surface of carbonized coffee ground (CWCG) was supported by subjecting the sample to ultrasonic treatment in a polyvinyl alcohol solution for one hour, followed by one hour of baking at 120 °C. Ultrasonic treatment induces crosslinking reactions in the PVA solution. Crosslinking reactions alter the physical properties of PVA and enhance the hydrophobicity of the material.

### Characterization of the PVA modified carbonized coffee ground

Scanning electron microscopy (SEM-EDX 6610LV), Energy dispersive X-ray spectroscopy (EDX) and Fourier Transform infrared spectroscopy (FT-IR 6600) were employed to analyze the raw waste coffee ground (WCG), carbonized waste coffee ground (CWCG) and modified carbonized waste coffee ground (MCWCG). The mass loss and thermal stability of the prepared material was analyzed using TGA (perkin elmer 8000). X-Ray diffractometer (Bruker D2 Phaser) was also employed to observe the crystallinity and crystal structure of the prepared materials. A contact angle goniometer (Osilla) was used to measure the contact angle (static) for both CWCG and MCWCG. IR thermometer (HT-822) was applied to measure the temperature profile. The light absorption of the material was measured by UV–Vis–NIR (Lambda 35) spectrometer. The solar irradiation power was recorded using a portable solar power meter (TM-207, Taiwan).

### Experimental investigation for vapor generation and collection

The vapor generation test was performed on a sunny day starting from 12:00 PM at noon. The ambient temperature was 27 °C and the experiment took 3 h. Thirty milligrams of the MCWCG were made to float on vessels containing 35 g distilled water. The change in mass was recorded using an analytical scale connected to a computer. The humidity of the day was 40–60% during the vaporization experiment.

**Figure 2:**
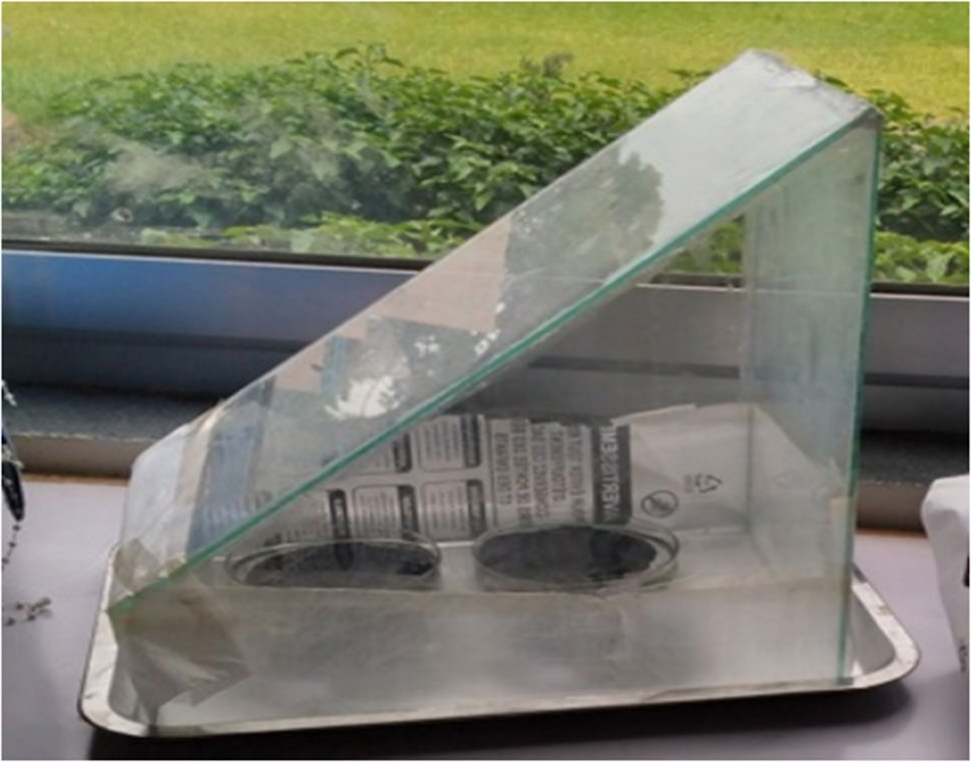
Experimental setup for vapor generation and collection.

To collect the evaporated water and characterize it, the vaporization experiment was conducted for three consecutive peak hours of solar power from 12:00PM to 3:00PM. To check the durability of the photothermal material, the steam generation experiment was continued for 5 consecutive days. For the steam collecting experiment, a light transparent glass with a 45° slope was constructed (Fig. 2). The glass cover’s inclination lets the condensed water flow down without mixing with the unpurified water.

The materials' photothermal conversion efficiency was estimated using the following equation^29^;

1 $$\eta = m {h}_{LV}/ I$$

where *m* is a steady state water vaporization rate (kg m^−2^ h^−1^), *h*_*LV*_ is the phase change total enthalpy change, and *I* is solar irradiation power density, which is (980 Wm^−2^)^29^.

*h*_*LV*_ is latent heat of vaporization, which can be estimated as the following equation^47^;

2 $${h}_{LV}= \Delta hvap + Cp\Delta T$$

where *Cp* is the specific heat capacity of water (4.2 kJ/kg K), and *h*_*vap*_ is the latent heat of vaporization of water at atmospheric pressure and *ΔT* is the change in temperature.

## Result and discussion

### Photothermal material preparation and characterization

**Figure 3:**
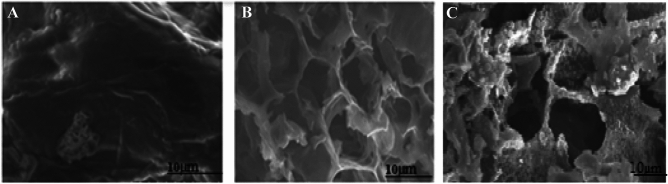
Morphological characteristics of raw waste coffee ground (**A**), carbonized waste coffee ground (**B**) and ZnCl2 modified carbonized coffee ground (**C**).

Coffee ground, carbonized coffee ground, and modified carbonized coffee ground were all imaged using a Spectro Electron Microscopy (SEM) (Fig. 3). As can be seen from the images below, the coffee ground has a porous structure (Fig. 3A), which indicates that it can be used for the removal of certain pollutants by adsorbing them on its porous surface. The carbonization is intended to enhance the surface characteristics and porosity for better pollutant adsorption (Fig. 3B). The carbonized waste coffee ground was further activated by ZnCl_2_ to enhance the surface (Fig. 3C). The further application of PVA did not significantly affect the surface structure of the carbonized coffee ground.

**Figure 4:**
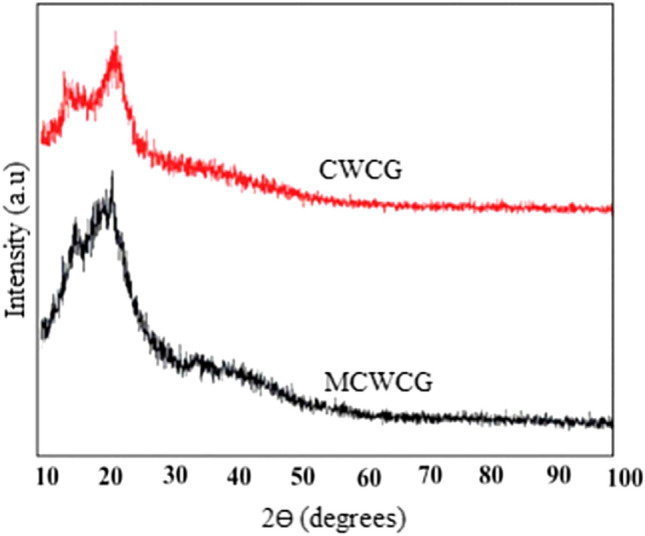
XRD analysis of carbonized waste coffee ground and modified carbonized waste coffee ground.

The crystallinity of the carbonized waste coffee ground (CWCG) and modified carbonized waste coffee ground (MCWCG) was analyzed using XRD (Fig. 4). As can be seen from the graph, both CWCG and MCWCG exhibit similar XRD patterns. The two crystalline regions in the XRD analysis pattern of the samples show two broad peaks at the 2Ɵ values of 18.4° and 22.2°. This is due to the crystal planes of β-crystal phase structure, which is also clearly seen during the initial stage of TGA analysis (Fig. 3). This indicates that the existence of strong hydrogen bonds (interaction) between the micro-structures resulted in high tensile strength as well as minimal accessibility for chemical attack. The presence of other organic/inorganic and hemicellulose in both samples shows the existence of an amorphous structure, which implies easy degradability and vulnerability to chemical attacks^48^.

**Figure 5:**
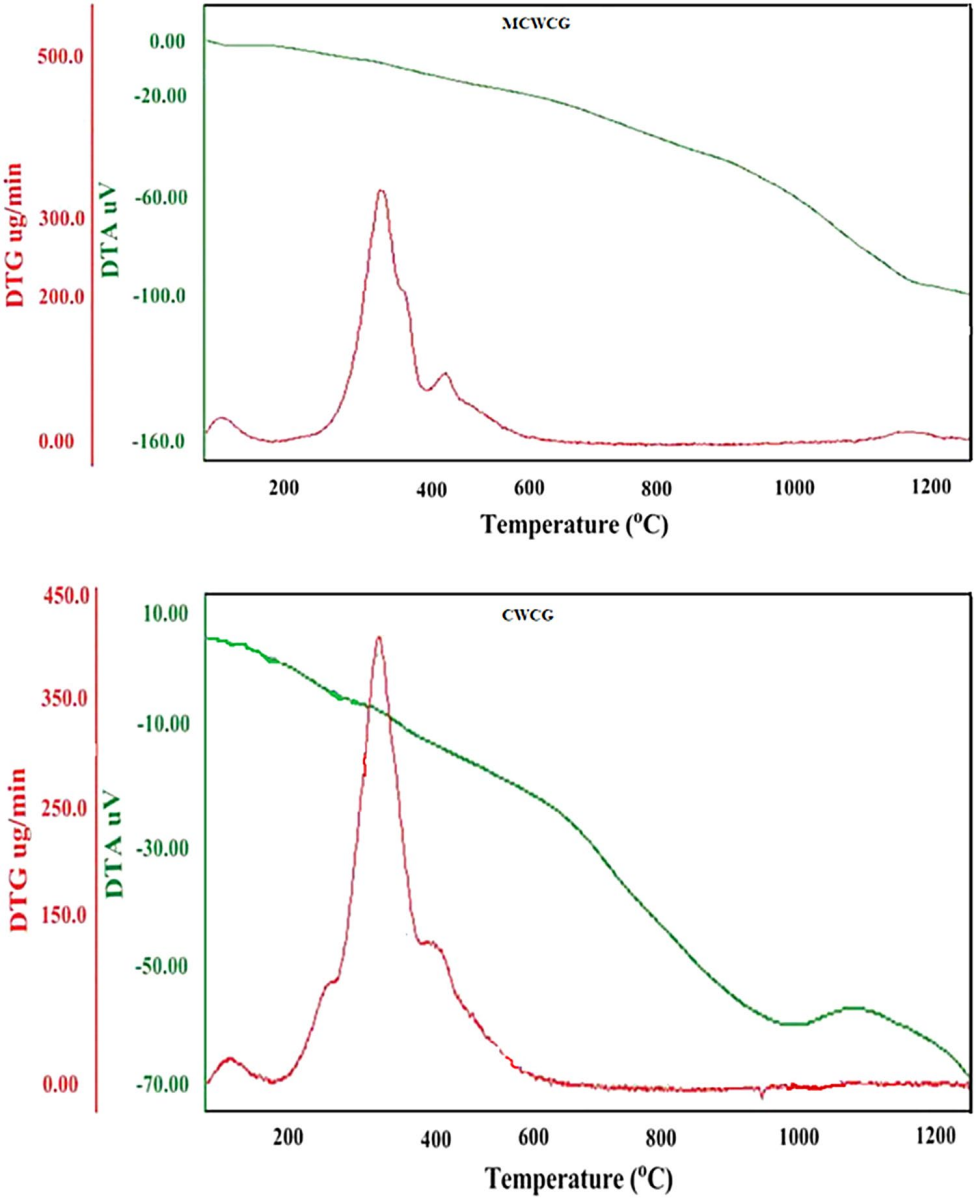
TGA and DTA of CWCG and MCWCG.

As can be seen from Fig. 5 below, both samples show a tri-stage decomposition until they reach 1200 °C, and for both CWCG and MCWCG, the decomposition curve is nearly similar. Mass reduction (4–6%) was observed at the beginning up to 60 and 75 °C, respectively, which is attributed to the removal of embodied water molecules from both materials. This also indicates that the materials are relatively stable at these temperature ranges. Between the temperature ranges of 245–365 °C (for CWCG) and 240–380 °C (for MCWCG), fatty oils and polysaccharides have been removed, resulting in a mass loss of 42 and 50%, respectively. Finally, almost all the materials decomposed starting at 500 °C (CWCG) and 510 °C (MCWCG), and the mass loss was 35% and 39%, respectively. The above results imply that the material is thermally stable, and therefore it can be suitably used for solar-thermal water purification.

**Figure 6:**
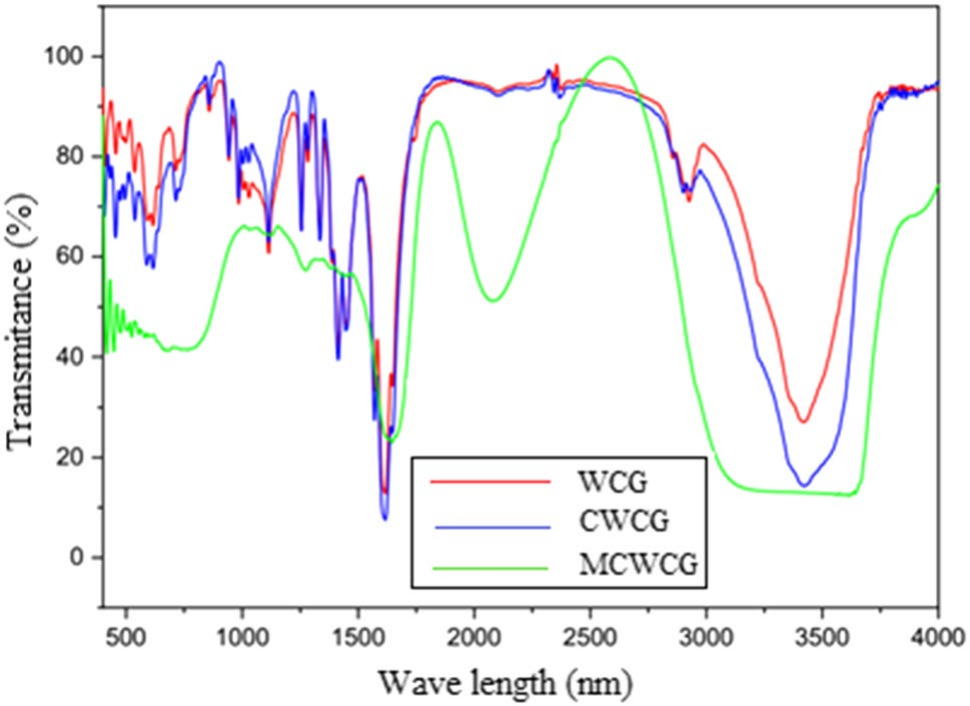
FTIR spectrum of WCG, CWCG, and MCWCG.

The prepared materials were further characterized by FT-IR spectroscopy to see their chemical characteristics (Fig. 6). Accordingly, the peak at 3400 cm^−1^ in the coffee ground profile was attributed to O–H stretching vibrations, which were largely produced by long-chain carboxylic acids. C–H vibrations have been linked to the peaks at 2700–3000 cm^−1^, implying the presence of alkane groups stretching^49^. The C–H bending of cellulose and hemicelluloses, as well as the C=O stretching from carboxylic acids and ester groups, were connected to the complicated peaks that occur between 1000 and 1700 cm^−1^. This could point to the presence of organic carbon in waste coffee grounds. C–H bending, notably related to aromatic ring compounds, was assigned to the broadband range of 600–1000 cm^−1^. During the manufacture of carbonized coffee ground, the peaks at 1000–1750 cm^−1^, 2700–3000 cm^−1^, and 3400 cm^−1^ were drastically reduced, indicating that the functional groups of C=O, C–H, and O–H were broken^50^. The peak shifting indicate that the carbonization procedure considerably increased broadband light absorption, which is important for achieving a high solar thermal conversion. This is a necessary step in achieving the goal of developing effective photothermal materials for water purification.

**Figure 7:**
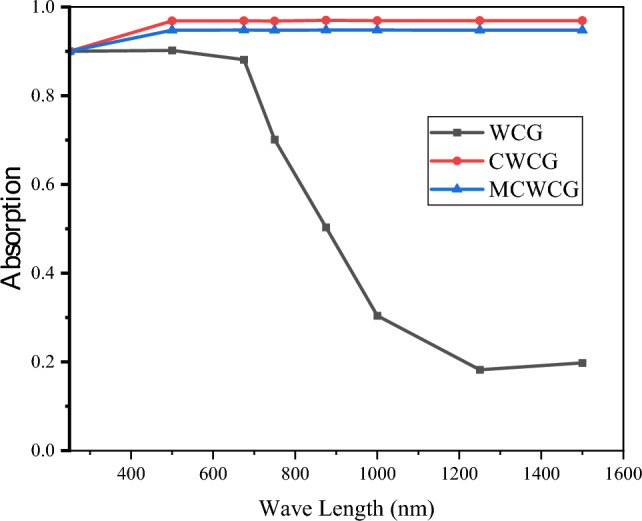
The UV–Vis–NIR absorption spectra of the WCG, CWCG, and MCWCG samples.

Energy Dispersive X-Ray Analysis (EDX) was employed to analyze the elemental composition of the three materials individually. The two main elements found in the prepared photothermal material were oxygen and carbon. Initially, in the raw material (waste coffee ground), the carbon percentage was 60%, and carbonization enhanced the carbon percentage to more than 90%, which can be attributed to the removal of oxygen compounds. However, the addition of ZnCl_2_ and PVA as a modifier decreased the carbon percentage to 86%, and a Zn and Si residual was observed in the modified photothermal material. It is clearly indicated that the surface modification by ZnCl_2_ was successful. Absorption spectra of the three materials (waste coffee ground (WCG), carbonized waste coffee ground (CWCG), and ZnCl_2_-modified waste coffee ground (MCWCG)) were performed to investigate whether the carbonization or ZnCl_2_-modification had contributed to the light absorption characteristics of the materials as shown in Fig. 7. It is revealed that the broadband for light absorption was significantly increased, which indicates that high solar thermal conversion can be achieved.

In a photothermal vaporization system, the materials should not sink into the water, hence, the self-floating characteristics of MCWCG are very important. If the MCWCG is immersed in water, it is very challenging for the sunlight to penetrate to the material. This is due to the higher heat capacity of water, which protects the conversion of heat on the surface of material. That is why PVA-modification was required to create hydrophobic characteristics and, hence, self-floatation. The contact profile of the water droplet was checked to see the wettability of the CWCG and MCWCG by a contact angle goniometer. The hydrophobicity of the CWCG was attained at a contact angle of 65°. However, after 25 min, all the CWCG sank down in the cylinder. However, the contact angle of MCWCG was 145°, which assures that PVA modification was effective in enhancing the hydrophobicity of carbonized waste coffee ground and hence self-flotation.

The prepared materials were analyzed using an IR thermometer to see the temperature profile under sunlight irradiation of 980 Wm^−2^. It was observed that the sun heated the interface of air and water. At the beginning, before the light illumination, the temperature of MCWCG was 25 °C. The interface temperature instantly increased to 36 °C in 6 min, which indicates that MCWCG can absorb solar irradiation effectively and is able to convert heat energy through the increased temperature at the interface of air–water, hence an efficient evaporation rate can be possible.

### Experimental investigation of the vaporization of lake water using the novel prepared material for water purification

The prepared material is then experimentally tested and validated. During the experiment, two main factors were considered, i.e., the amount of modifier (PVA) and the carbonized waste coffee ground itself. The experimental data of evaporation rates were optimized by using design expert software as shown in Table 1.

**Table 1:** The experimental data of evaporation rates.

Run	Factor-1 Amount of MCWCG (g/m^2^)	Factor-2 Conc. of PVA (mg/mL)	Response Vaporization rate (kg/m^2^h)
1	50	30	1.12
2	75	30	1.05
3	75	30	1.13
4	50	10	1.03
5	75	50	1.11
6	75	30	1.13
7	75	10	1.10
8	75	30	1.11
9	50	50	1.03
10	100	10	1.04
11	100	30	1.07
12	100	50	1.05

The adequacy and significance of the quadratic model was justified by the analysis of variance (ANOVA) as shown in Table 2.

**Table 2:** ANOVA for Quadratic mode (Response: Rate of vaporization).

Source	Sum of squares	Df	Mean square	F-value	*p*-value	
Model	0.0184	5	0.0037	81.08	< 0.0001	Significant
A-amount of AC	0.0004	1	0.0004	9.19	0.0191	
B-Conc. of PVA	0.0001	1	0.0001	1.47	0.2647	
AB	0.0000	1	0.0000	0.5511	0.4820	
A^2^	0.0115	1	0.0115	254.52	< 0.0001	
B^2^	0.0011	1	0.0011	23.52	0.0019	
Residual	0.0003	7	0.0000			
Lack of Fit	0.0000	3	0.0000	0.1787	0.9056	Not significant
Pure Error	0.0003	4	0.0001			
Cor Total	0.0187	12				

**Figure 8:**
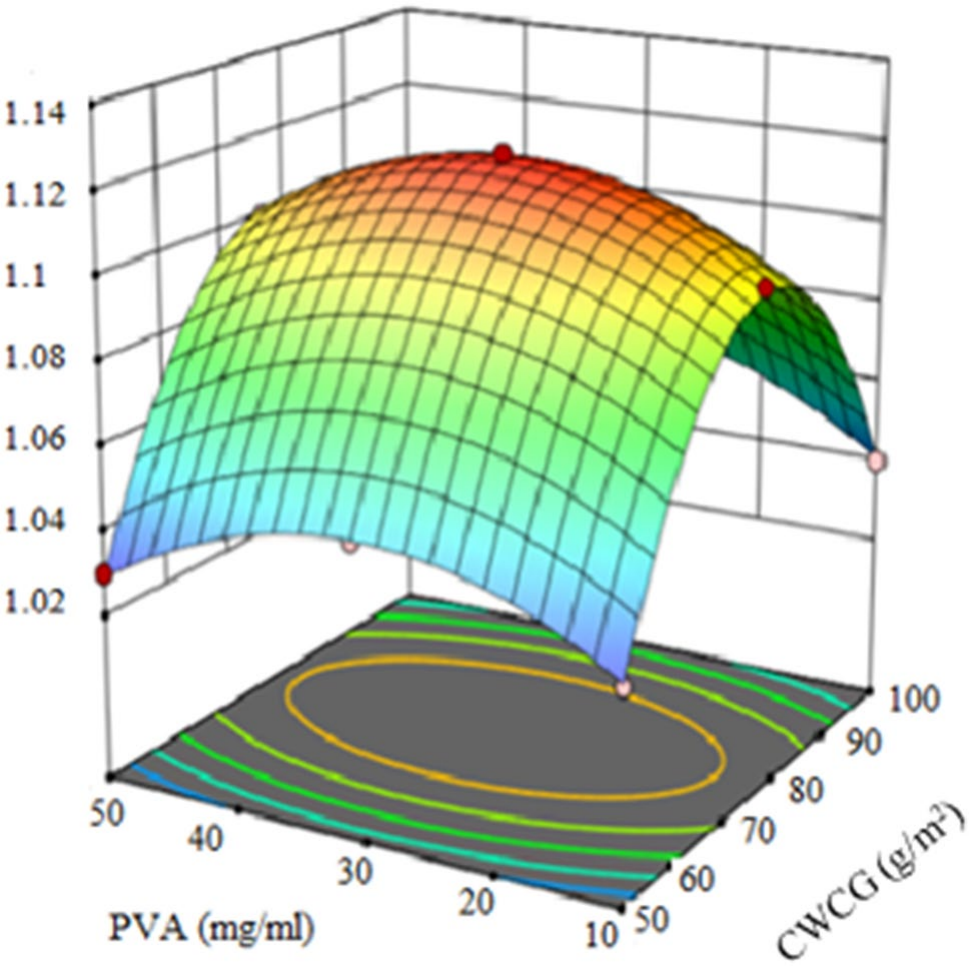
Interaction effects of PVA and CWCG concentration on water evaporation rate.

The Model F-value of 81.08 implies the model suggested by the software was significant. The *p*-value of < 0.0001, which was < 0.05, also confirms that the model was valid. Though the amount of MCWCG and the PVA concentration were significant factors, it was observed that the amount of MCWCG was more important than the concentration of PVA (Fig. 8).

The water vaporization rate with the MCWCG was dependent on the amount deposited per unit area. The highest rate was obtained when the photothermal layer was prepared via depositing 76.51 g/m^2^. This indicates that excessively thick or thin photothermal layers could retard steam generation by insufficient water uptake or by low photothermal conversion. Indeed, improvements in the water wettability of originally hydrophobic photothermal material are essential for effective solar-to-steam generation. Thus, water-soluble polymers were introduced during the deposition process. Specifically, PVA was selected with its high capacity of reducing the latent heat of evaporation of water and its adhesive property was also important for the deposition.

The interaction response of the prepared material amount and PVA concentration were analyzed and presented using interaction graph (Fig. 8). This analysis aimed to find out the significant effects of the process parameters amount of CWCG and PVA concentration on the rate of vaporization. 3D response surface contour plot is used to show the effect of the two factors on the responses. The water evaporation rate was increased after the PVA treatment. It was found that 30.9 mg/mL of PVA was suggested as the optimal concentration that exhibits the best performance of solar-to-steam generation. The water evaporation rate by MCWCG reached 1.12 kg/m^2^h under the illumination of 980 Wm^-2^ including the water evaporation rate without sunlight radiation. The water evaporation rate without sunlight radiation was measured to be 0.107 kg/m^2^h. The water evaporation rate was increased in the presence of MCWCG. When the PVA concentration exceeded 30.9 mg/mL, the water evaporation rate decreased slightly. The reason might be because the polymer molecules blocked the active intraparticle pores due to the swelling effect, and this resulted in retarded water uptake and decreased evaporation of water. The maximum rate of vaporization of 1.12 kg/m^2^h was observed at constant CWCG of 76.51 g/m^2^ and PVA of 30.9 mg/mL meaning that two factors were constant. Thus, the optimum amount of CWCG which was 76.51 g/m^2^, and the optimum amount of PVA which was 30.9 mg/mL solution were determined from the design expert software analysis. At those design points, the maximum rate of evaporation was 1.12 kg/m^2^h.

It was not significant in relation to the pure error, according to the Lack of Fit F-value of 0.18. A big Lack of Fit F-value had a noise probability of 90.56 percent of occurring. It was desirable for the model to fit despite the non-significant lack of fit as shown in Table 3.

**Table 3:** Fit Statistics (R^2^, Adjusted R^2^, Predicted R^2^).

Std. Dev	0.0067	R^2^	0.9830
Mean	1.08	Adjusted R^2^	0.9709
C.V. %	0.6214	Predicted R^2^	0.9615
		Adeq Precision	20.4295

The signal to noise ratio can be indicated by Adeq. precision and a ratio greater than 4 are desirable and it is for this case. The adjusted and predicted R^2^ values are in almost complete agreement and therefore, the model can be used to go through the design space.

**Figure 9:**
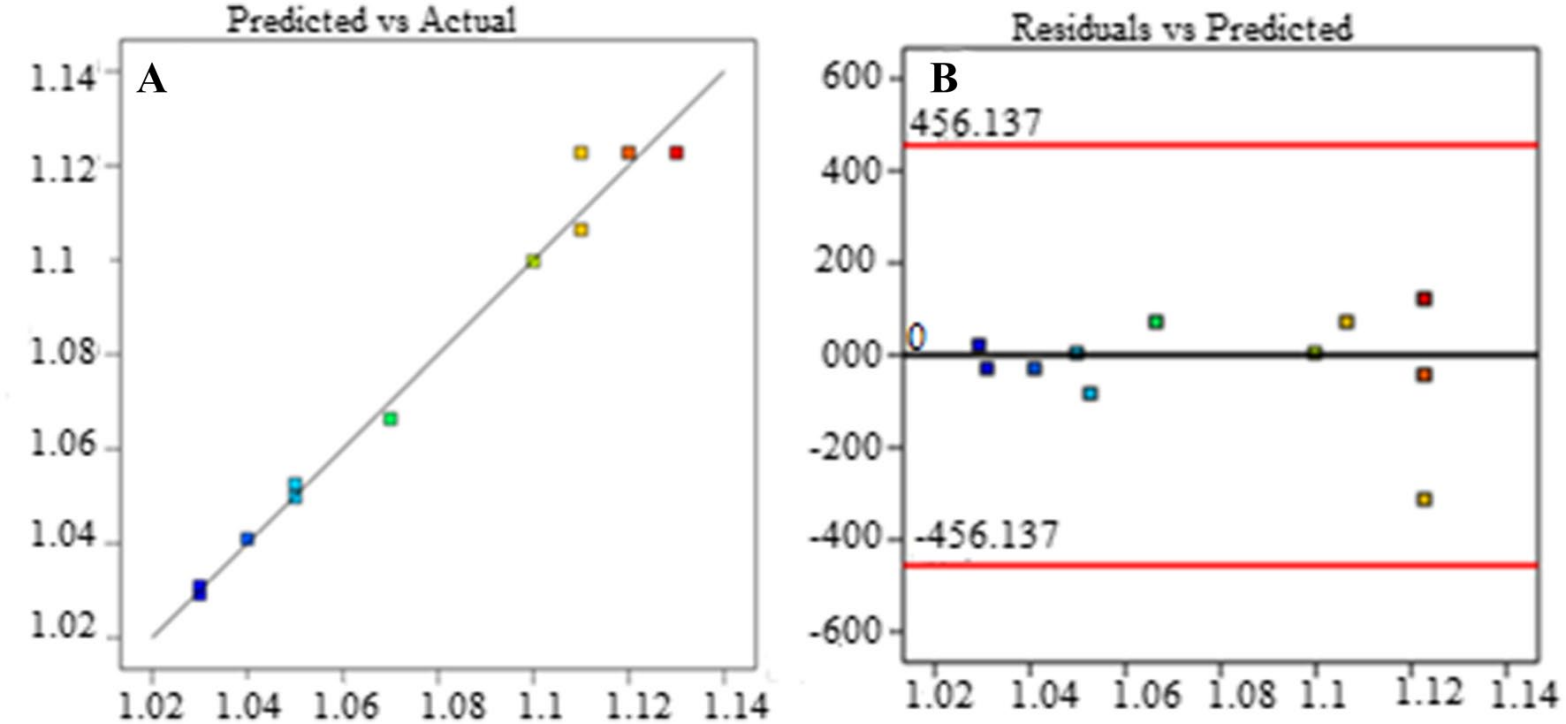
Predicted versus actual (**A**) and residuals versus predicated (**B**) vaporization rate analysis.

The predicted versus actual vaporization rate analysis as presented in Fig. 9 shows that the actual rate of vaporization is much closer to the predicted value. This implies the experimental work of vaporization was in the good truck and all results of the analysis were taken as correct. In addition, the objective of investigating the high rate of vaporization was achieved.

**Figure 10:**
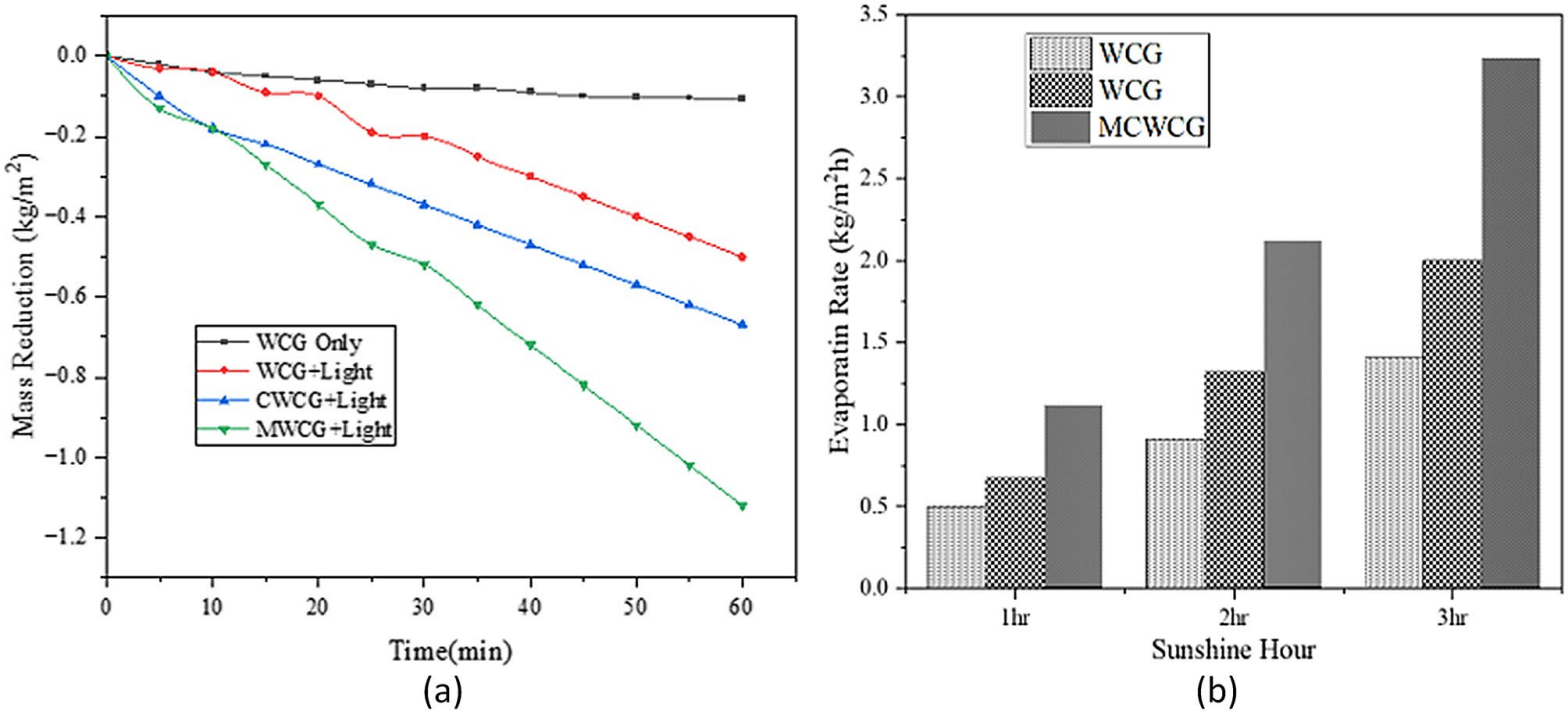
(**a**) Mass change of water profile by solar-to-steam generation for 1 h with 980 Wm^-2^ solar power; (**b**) Effect of sunshine hour and light on the rate evaporation.

The actual water evaporation rate was analyzed using apparent mass change (Fig. 10). The mass change was evaluated by keeping the material on the surface of the water and recording the weight loss against time. The vaporization performance was also analyzed using an apparent mass change method. The mass change was evaluated by keeping the material on the surface of the water and recording the weight loss against time (Fig. 10). The mass-loss rate of natural water without light irradiation was 0.107 kg/m^2^h. With 1 sun irradiation, the mass loss rate of water for the MCWCG was 1.12 kg/m^2^h, which was much greater than that of water (0.5 kg/m^2^h) and that of the AC/water system (0.67 kg/m^2^h).

The evaporation efficiency of the prepared photothermal material was calculated to be 74.5% under 980 Wm^-2^ illumination for one hour, which was higher than the value of the carbonized coffee ground steam generation materials of the previous study^51^. Under solar power irradiation of 980 Wm^−2^, the solar thermal efficiency for water was 32% and enhanced to 74.5% with the application of MCWCG. Therefore, MCWCG can perform better solar vaporization with great efficiency using solar power of 980 Wm^−2^. 

As the sun power increased somewhat over the course of the 5 days, the rate of vaporization likewise slightly increased. As the sun power increased somewhat over the course of the 5 days, the rate of vaporization likewise slightly increased. During the 5 consecutive days, as a slight increase in the solar power, there was also a slight increase in the rate of vaporization. As the slight decrease in the solar power, there was also a slight decrease in the rate of vaporization. Similar phenomena continued during the 5 days. Thus, the same photothermal material can be reused for several days without losing its performance and durability. The advantages of the carbonized coffee grounds-based photothermal over many other types of photothermal materials are cheap, good evaporation efficiency, and ease of production.

The prepared photothermal material performance can be considered as good as compared to the carbon-based materials (Table 4). Others prepared the material from virgin waste materials and therefore they can make their choice of parameter fixation based on their objectives. However, in this case, the proposed approach is the method of waste recycling and reuse as a new material.

**Table 4:** A comparison of different photothermal compounds made of carbon that are used for solar evaporation.

Materials	Solar intensity (Wm^-2^)	Evaporation rate (kg/m^2^h)
Carbonized tissue membrane	1000	0.86^23^
Carbon-nanotube-based floating solar still	1000	0.88^51^
Surface carbonized wood	1000	1.08^15^
Flame-treated wood	1000	1.05^52^
Hydrophobic carbonized coffee grounds	1000	1.05^51^
Carbon nanotube-modified flexible wood membrane	1000	0.95^23^
Carbonized pomelo peel particles composited with the Chitosan (CS) aerogel	1000	1.78^53^
ink-stained paper-black	1000	1.25^54^
Carbonized coffee grounds modified with ZnCl_2_/PVA (this study)	980	1.12

It further explored the utilization of MCWCG layers for water purification of the simulated lake water. Comparison of the levels of Cu, TDS and total coliform concentrations, turbidity, and total hardness of the water samples (N = 10) with the purified water using the photothermal material as shown in Table 5.

**Table 5:** Simulated untreated water and treated water with MCWCG test results as compared to the permissible limit.

Parameter	Untreated sample water	Treated water	Maximum permissible level of pollutant (WHO)
Turbidity (NTU)	100–130	3–15	5
Total hardness (as CaCO_3_) (mg/L)	70–100	35–68	300
TDS (mg/L)	55–85	15–42	1000
Cu (mg/L)	0–0.12	0–0.02	0.05
Total coliform bacteria (CFU/100 mL)	80–110	0.5–15	Not detected

From Table 5, it was found that the prepared photothermal material, MCWCG could produce clean water to meet the permissible limit of pollutants in terms of total hardness, TDS, Copper. Heavy metals like copper were able to be reduced by 83%; whereas 95.6% of the bacterial count was removed. The treated water also can be further upgraded by a simple method of sand filtration to achieve the WHO standard. Therefore, with further development and improvement of this technology, it will be challenging to apply lake water purification without chemical addition. Previous studies also reaffirm that the solar steam water purification apparatus may generate good drinking water from river water and lake water^30^.

## Conclusions

In this study a photothermal material from waste coffee grounds was prepared and modified for lake water purification. The prepared material was experimentally tested and validated by constructing a simple evaporation–condensation system. The developed material was able to purify simulated lake water significantly. The produced modified material had a photothermal conversion efficiency of 74% and could produce vapor at a rate of 1.12 kFg/m^2^h under 980 W/m^2^ irradiation at 1 sun. Pollutants like metal ions (Cu^2+^) and microbes were removed above 90%. Therefore, with further development the modified carbonized waste coffee grounds (MCWCG) can be implemented for water purification, and hence, contribute to alleviating water scarcity and reduce environmental pollution.

## Data Availability

All data is included in this article. There is no supplemental data available in this article.
